# Noninvasive Compartmental Pressure Assessment With iCare in Healthy Individuals of Different Ages

**DOI:** 10.1111/os.70078

**Published:** 2025-06-16

**Authors:** Jialiang Guo, Jianfeng Zhang, Kezheng Du, Bo Shi, Weichong Dong, Yingze Zhang, Zhiyong Hou

**Affiliations:** ^1^ Department of Orthopaedics Hebei Medical University Third Hospital Shijiazhuang China; ^2^ Department of Pharmacy The Second Hospital of Hebei Medical University Shijiazhuang China; ^3^ Engineering Research Center of Orthopedic Minimally Invasive Intelligent Equipment, Ministry of Education Shijiazhuang China; ^4^ Key Laboratory of Biomechanics of Hebei Province Shijiazhuang Hebei China; ^5^ NHC Key Laboratory of Intelligent Orthopeadic Equipment (Hebei Medical University Third Hospital) Shijiazhuang China; ^6^ Chinese Academy of Engineering Beijing China

**Keywords:** acute compartment syndrome, compartmental pressure, fascia, noninvasive measurement

## Abstract

**Objective:**

Acute compartment syndrome is a major orthopedic emergency due to elevated pressure in the closed muscle compartment, and prompt evaluation and fasciotomy are always needed. However, the gold standard indicator of fasciotomy is still under debate. To date, few studies have investigated the variations in compartmental pressure at different locations in people of different ages. The aim of the research was to compare compartmental pressure among different age groups and measurement locations.

**Methods:**

A total of 154 healthy individuals including 106 males and 48 females over 18 years (46.8 ± 14.0 years) were enrolled between January 2020 and December 2021, and classified into five age groups: Group I = 18–30 years; Group II = 31–40 years; Group III = 41–50 years; Group IV = 51–60 years; and Group V ≥ 61 years. Six measurement locations (lower, middle, and upper points, 6 points) were selected to assess pressure variations in the anterior compartment and posterior superficial compartment with the iCare device, which calculates biomechanical properties based on the tissue's inherent response. Differences in pressure among the five age groups and six measurement locations (three for anterior compartment, three for posterior superficial compartment) were examined. One‐way ANOVA and LST tests were used to conduct comparisons among five independent age groups.

**Results:**

In the same measurement location, the compartmental pressure in Group V at the upper anterior (anterior fascial compartment) measurement location was increased compared with that in Groups I, III, and IV. However, the compartmental pressures at the middle anterior and posterior measurement locations were almost comparable among the five different age groups. In the same age group, the compartmental pressure was more inclined to be lower at the upper anterior measurement location in Groups I–IV. However, no significant differences were observed for other measurement locations.

**Conclusions:**

The measurement results demonstrated comparable compartmental pressure in the fascial compartment at most measurement locations. The fascia, which forms the limb compartment, may play a role in pressure release or redistribution after injury or fracture due to its function and unique or interconnected structure.

## Graphical abstract

The aim of the research was to compare compartmental pressure among different age groups and measurement locations. Healthy individuals who were older than 18 years were enrolled and classified into five age groups. Six measurement locations (lower, middle and upper points, 6 points) were selected to assess pressure variations in the anterior compartment and posterior superficial compartment with the iCare device, which calculates biomechanical properties based on the tissue's inherent response. The measurement results demonstrated comparable compartmental pressure in the fascial compartment at most measurement locations. The fascia, which forms the limb compartment, may play a role in pressure release or redistribution after injury or fracture due to its function and unique or interconnected structure.

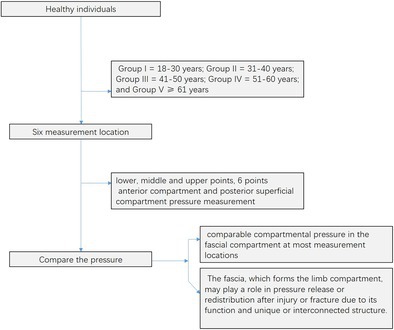

## 1 Introduction

Acute compartment syndrome (ACS) is a major orthopedic emergency due to elevated pressure in the closed muscle compartment, and prompt evaluation and fasciotomy are always needed [1, 2]. ACS is a relatively common complication that is limb threatening and can result in devastating morbidities and mortality. Failure to perform an urgent surgical intervention once recognized can lead to irreversible tissue damage within hours of onset. However, the myofascial release law, which was proposed in our previous research, illustrated that when blistering occurred, alleviation of compartment symptoms such as pain and weakness was observed, resulting in a reduced risk of ACS [2, 3].

Currently, ACS can be diagnosed by the invasive or noninvasive measurement of compartmental pressure, and disparity between diastolic and compartmental pressure has been established as the gold standard indication for fasciotomy (diastolic pressure minus compartmental pressure < 30 mmHg) in the clinic [4, 5]. However, the measurement results will be affected by limb position, and the measurement modalities can carry the risk of infection and bleeding, especially invasive modalities [6, 7]. Furthermore, the result can vary according to changes in the measurement position or location [8]. A study of knee cadaveric specimens concluded that even when the proper technique was used, 40% of the measurement results were 5 mmHg larger than the actual pressure. Second, no standard location for the pressure measurement, for example, closer to or further away from the fracture (outside the zone of injury), has been established to obtain more representative measurements of compartmental pressure [9]. Nudel found that the pressure distribution was not uniform in the myofascial compartment and that the compartmental pressure adjacent to a seriously damaged artery was significantly higher than the critical value; however, the pressure measured at locations far from the injured artery was substantially lower than the threshold, even 2 h after injury [10]. Other studies found the compartmental pressure to be highest within 5 cm of the fracture site and recommended this as the measurement area [9, 11]. However, as a self‐contained structure of the lower limb, the pressure of the compartment at different locations should be comparable. In addition, differences in structural characteristics such as muscle tissue volume and fascial toughness will also affect the pressure measurement results in people of different ages.

To date, the compartmental variations among different locations in people of different ages have not yet been clearly defined. Few experiments on trends in compartmental pressure variations allowing comparisons among young and old individuals have been reported. In this research, the compartmental pressure was measured at different locations of the lower limb in healthy individuals and aims to (i) reveal the potential mechanism of compartment pressure redistribution; (ii) testify whether the fascia is a functional organ; and (iii) illustrate the advantage and clinical application potential of noninvasive compartment pressure measurement with Icare.

## 2 Materials and Methods

### 2.1 Demographic Data

Electronic medical records from healthy volunteers with normal lower limbs between January 2020 and December 2021 were extracted from the hospital medical records department. The retrospective study was approved and performed in accordance with the ethical standards of the 1964 Declaration of Helsinki. Formal ethical approval was received from the Regional Ethics Committee of our hospital (S2020‐024‐1). The registered clinical trial was NCT04529330 (27/08/2020).

Healthy volunteers older than 18 years who provided written informed consent were eligible for inclusion. The subjects enrolled were classified into five different age groups: Group I = 18–30 years; Group II = 31–40 years; Group III = 41–50 years; Group IV = 51–60 years; and Group V ≥ 61 years. The exclusion criteria were as follows: previous fracture or surgery; history of compartment syndrome; limb anomaly; or general muscle disorder affecting the lower leg. The mean age of the enrolled subjects (106 males, 48 females) was 47 ± 14.0 years (range: 18–84 years). The activity level was calculated with the Barthel index, and muscle diameter, which was measured in the middle region of the lower limb, was also included in this research.

### 2.2 Pressure Measurement

Compartment normative pressures were measured with a device called iCare (TA01, iCare Oy, Vanda, Finland), which is a handheld device that calculates biomechanical properties based on the tissue's inherent response, and the device does not affect compartmental pressures when positioned and pressed against the skin [12]. It has been proved to have potential application value in clinical application for noninvasive measurement of compartment pressure [13]. Each participant was placed in the supine position on an examination table and asked to rest and relax comfortably for 5 min. The undressed lower limb was positioned flat on the bed, and the knee was extended. The iCare couples with a disposable probe that is propelled from the tonometer toward the skin (0.25–0.30 m/s) by an electrical‐pulse generator. The speed of the probe as it moves back into the device causes a change in the magnetic field, which is captured by the tonometer, subjected to calculation, and converted into a pressure value (mmHg). Demographic data such as ages, gender, and so on were recorded at the beginning of the test. All examinations were conducted by one examiner to exclude potential observer bias.

### 2.3 Measurement Position

The anterior and posterior superficial compartments of the right lower limb were chosen to assess the variations in compartmental pressure among different measurement locations. The lower limb was equally divided into three parts with the same width in each individual, and the measured sites (lower, middle and upper points, six points) were marked on the medial (posterior superficial fascial compartment) and lateral (anterior fascial compartment) sides for repeated compartment measurements. The upper two points (anterior‐upper, AU and posterior‐upper point, PU) were located at the middle line of the upper third, which was located just 3 cm above the ankle mortise. The middle two points (anterior‐middle, AM and posterior‐middle point, PM) were located at the middle line of the middle third, which was located at the middle of the tibia shaft. The lower two points (anterior‐lower, AL and posterior‐lower point, PL) were located at the middle line of the lower third, which was located just 3 cm below the tibial plateau. Six measurements were obtained in the same location at 5‐s intervals to minimize any potential measurement variation. The six data points were recorded, and their average value (three times) was selected as the final result. Finally, the AU, PU, AM, PM, AL, and PL fascial compartmental pressures were recorded.

### 2.4 Statistical Analysis

Continuous data are presented as the means and standard deviations. Homogeneity of variance for continuous variables was evaluated with the Levene test. One‐way ANOVA and LST tests were used to conduct comparisons among 5 independent age groups. Bonferroni was used as the *p* value correction method. For all analyses in this research, statistical significance was set at *p* < 0.05 before data analysis. All analyses were conducted using SPSS version 22.0 (IBM Corp, Armonk, NY).

## 3 Results

### 3.1 Baseline Data

A total of 154 healthy individuals including 106 male and 48 female who were older than 18 years (46.8 ± 14.0 years) were enrolled. There were 19 subjects in Group I (male 13, female 6), 29 in Group II (male 13, female 6), 34 in Group III (male 26, female 8), 39 in Group IV (male 31, female 8), and 28 in Group V (male 15, female 13).

### 3.2 Compartmental Pressure in Different Age Groups

**FIGURE 1:**
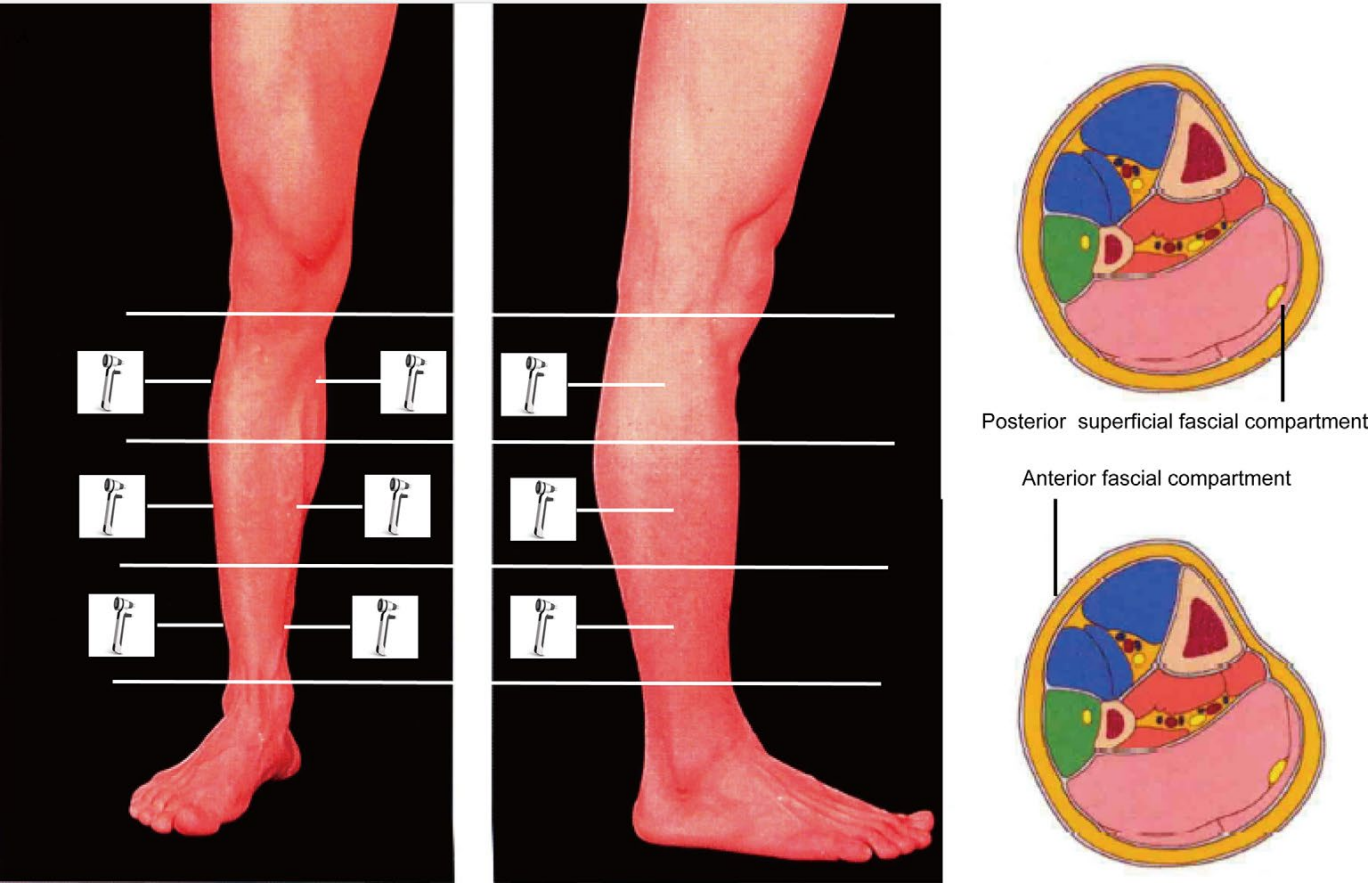
The measurement location of lower limb intracompartment pressure. The lower limb was divided into three identical parts, and the sites (upper, middle, and lower points) for the anterior and posterior superficial compartments.

**FIGURE 2:**
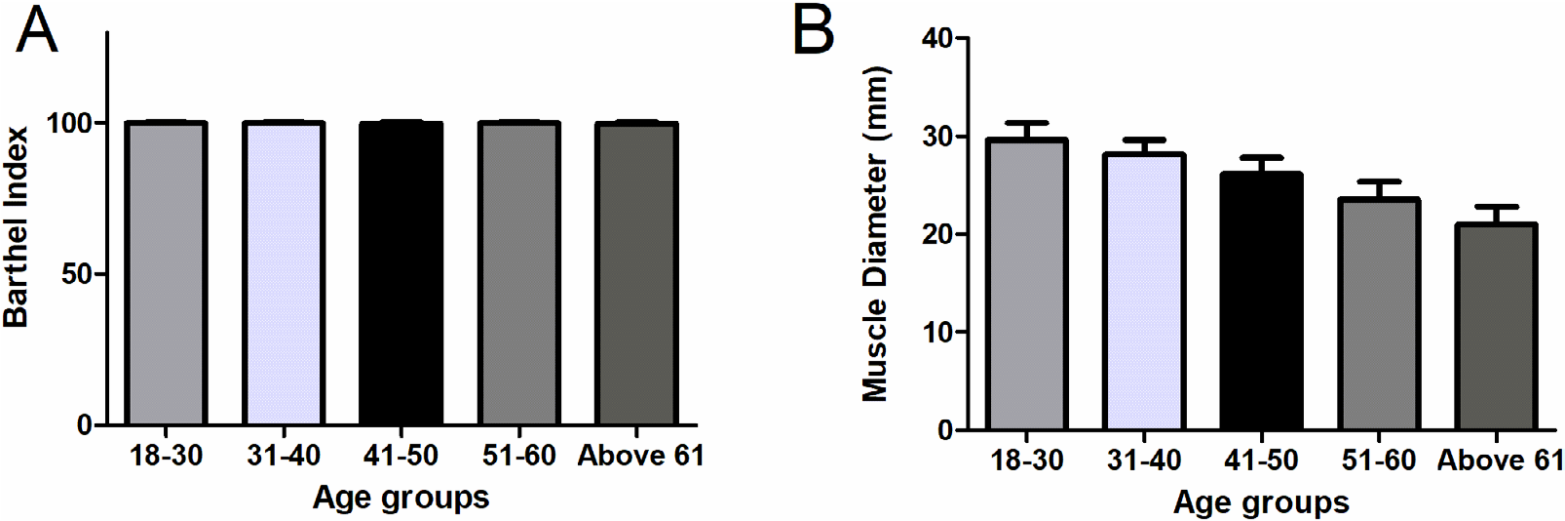
The activity level and muscle diameter comparison in different ages groups. (A) The Barthel index comparison. (B) The muscle diameter in five age groups.

The measurement position and the compartment measured was illustrated in Figure 1. No significant differences were illustrated about the activity level, but the muscle diameter can be found a significant differences in different age groups (Figure 2). There was a significant increase in the compartmental pressure in Group V (21.36 ± 3.00) at the upper anterior (anterior fascial compartment) measurement location compared with that in Group I (19.5 ± 3.27, *p* = 0.038), Group III (18.23 ± 3.20, *p* < 0.001), and Group IV (19.80 ± 3.15, *p* = 0.036). An obvious increase in the compartmental pressure in Group II (20.12 ± 2.56, *p* < 0.001) and Group IV (19.80 ± 3.15, *p* = 0.03) was also observed compared with that in Group III. A similar trend was also observed at the upper medial (posterior superficial fascial compartment) measurement location, with a significant increase in the compartmental pressure in Group V (21.84 ± 5.76, *p* = 0.001) compared with that in Group I (19.40 ± 3.61, *p* = 0.025), Group II (18.88 ± 3.66, *p* = 0.003) and Group III (18.66 ± 2.91, *p* = 0.001).

**FIGURE 3:**
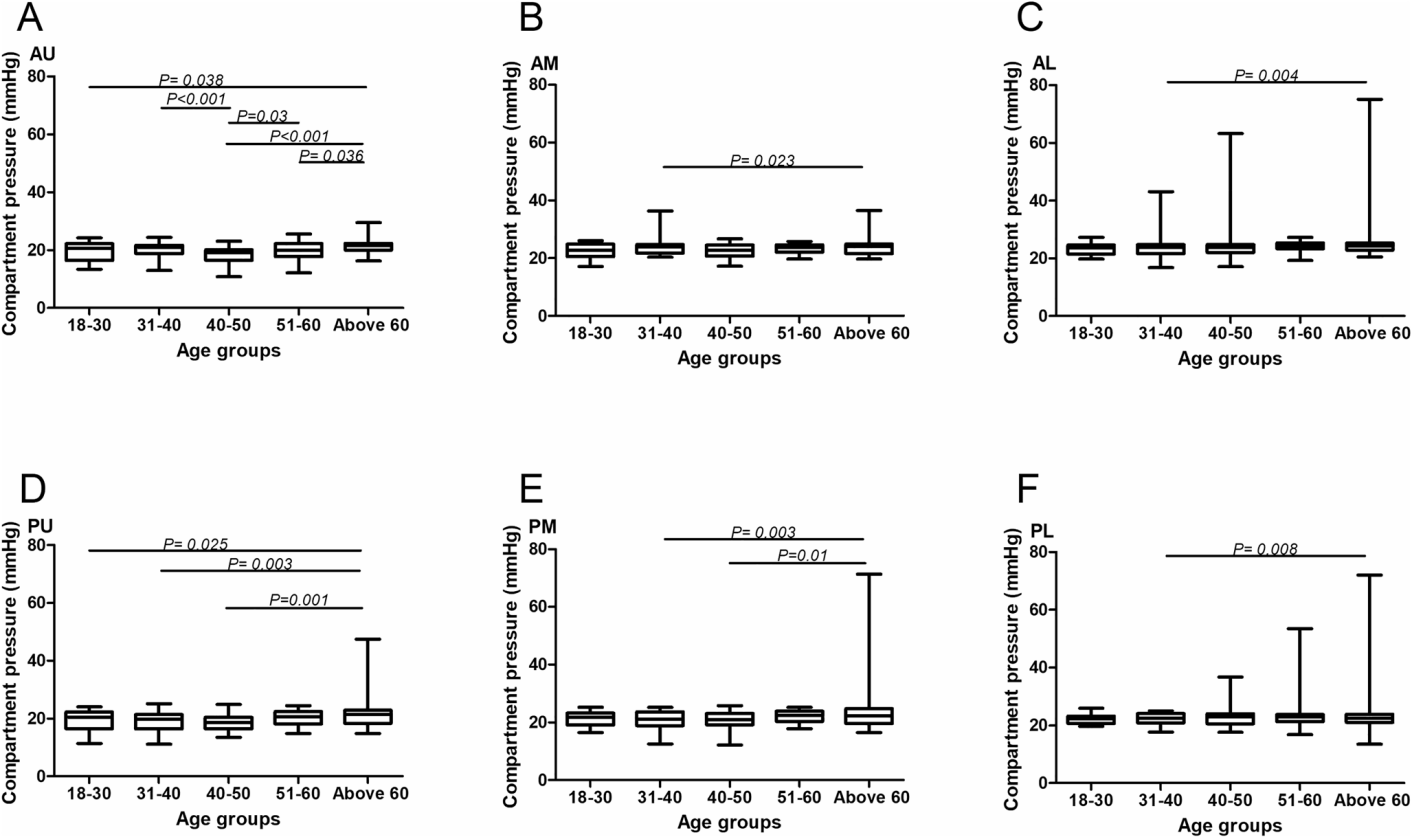
Pressure measurement result comparison in different age groups. (A, D) Pressure results comparison on anterior and posterior upper measurement location. (B, E) Pressure results comparison on anterior and posterior middle measurement location. (C, F) Pressure results comparison on anterior and posterior lower measurement location.

The compartmental pressures at the middle anterior and posterior measurement locations were comparable except between Groups V and II (anterior, 23.86 ± 3.14 vs. 23.75 ± 2.90, *p* = 0.023; posterior, 23.85 ± 9.74 vs. 20.89 ± 3.12, *p* = 0.003) and Groups V and III (posterior, 23.85 ± 9.74 vs. 20.75 ± 3.13, *p* = 0.01). Furthermore, the compartmental pressures at the lower anterior and posterior measurement locations were comparable, except for those in Groups V and II (anterior, 26.31 ± 9.97 vs. 24.61 ± 7.14, *p* = 0.004; posterior, 25.05 ± 12.12 vs. 22.66 ± 3.39, *p* = 0.008) (Figure 3).

### 3.3 Compartmental Pressure at Different Measurement Locations

**FIGURE 4:**
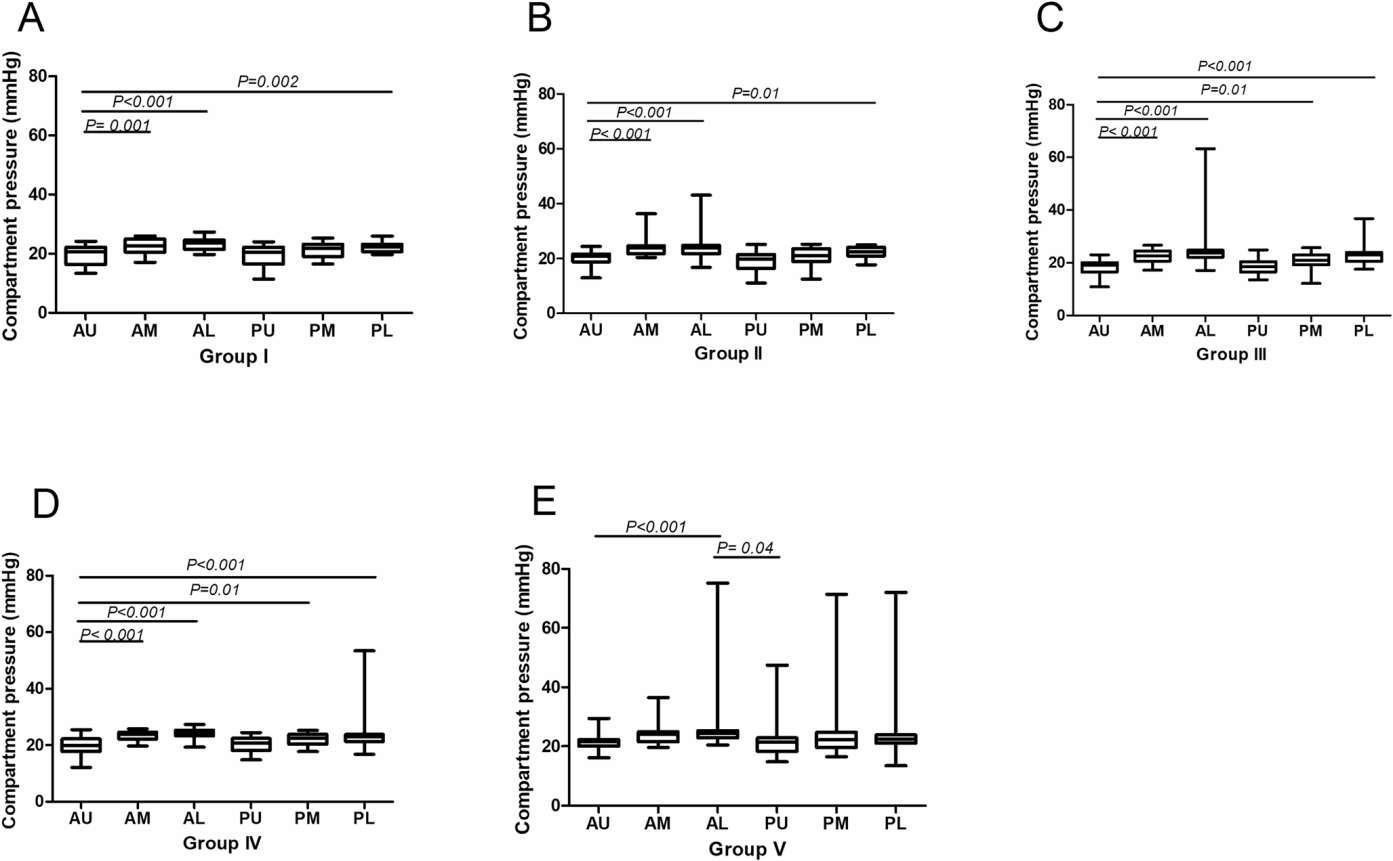
Pressure measurement result comparison in different measurement locations. (A) Pressure results on the second day after injury in the 18–30 age group. (B) Pressure results on the second day after injury in the 31–40 age group. (C) Pressure results on the second day after injury in the 41–50 age group. (D) Pressure results on the second day after injury in the 51–60 age group. (E) Pressure results on the second day after injury in the ≥ 61 age group.

**FIGURE 5:**
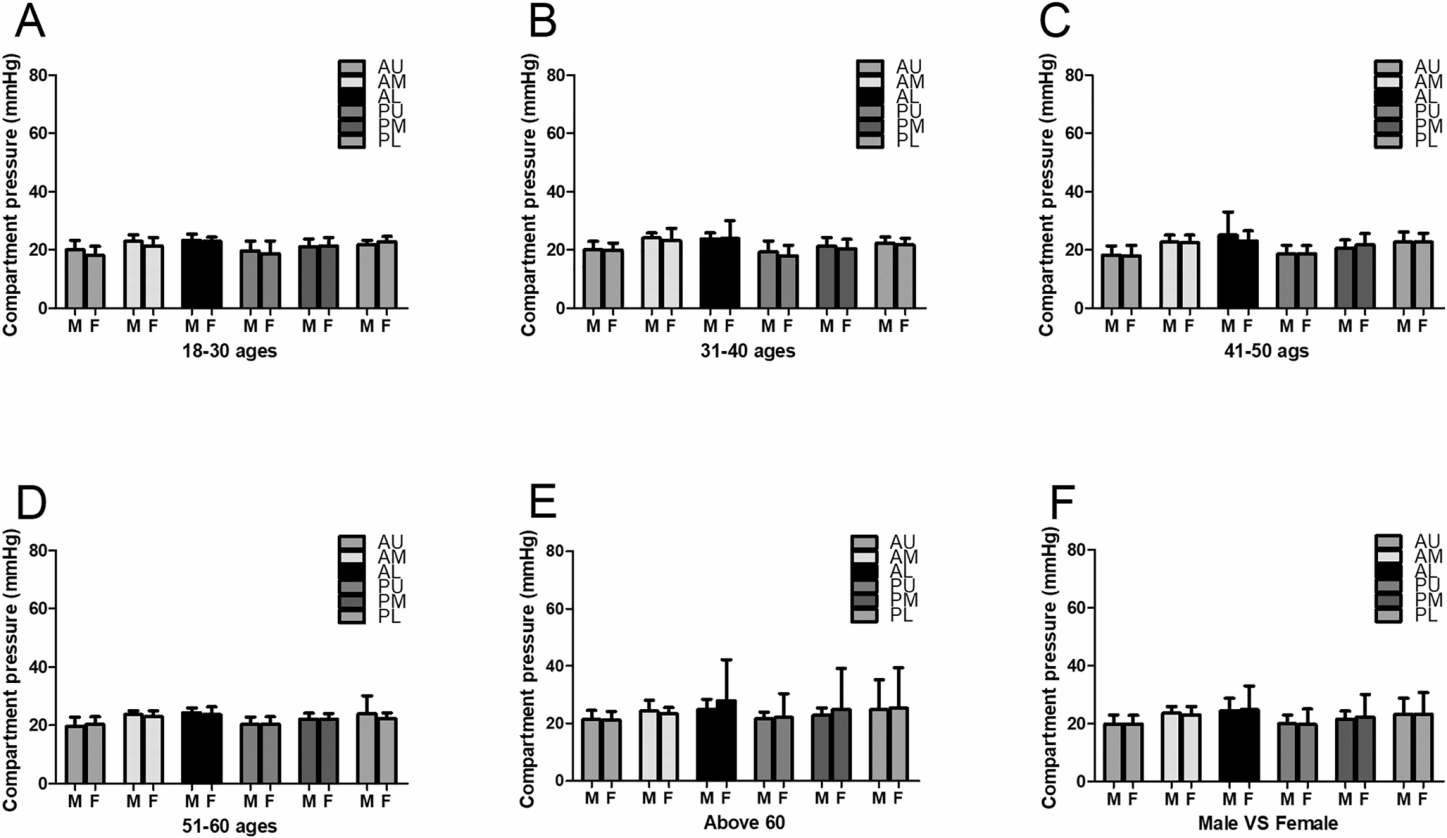
Pressure measurement result comparison in different sex. (A) Pressure results comparison in the 18–30 male and female. (B) Pressure results comparison in the 31–40 male and female. (C) Pressure results comparison in the 41–50 male and female. (D) Pressure results comparison in the 51–60 male and female. (E) Pressure results comparison in the ≥ 61 male and female. (F) Pressure results comparison in male and female.

In Groups I and II, the compartmental pressure was significantly lower at the upper anterior measurement location (Group I, 19.50 ± 3.27; Group II, 20.12 ± 2.56) than at the middle anterior (Group I, 22.50 ± 2.56, *p* = 0.001; Group II, 23.75 ± 2.90, *p* < 0.001), lower anterior (Group I, 23.20 ± 1.90, *p* < 0.001; Group II, 23.82 ± 3.99, *p* < 0.001), and lower posterior (Group I, 22.20 ± 1.56, *p* = 0.002; Group II, 22.11 ± 2.21, *p* = 0.01) locations. However, no significant differences were observed among other measurement locations. In Groups III and IV, the compartmental pressure was significantly lower at the upper anterior (Group III, 18.23 ± 3.20; Group IV, 19.80 ± 3.15) measurement location than at the middle anterior (Group III, 22.67 ± 2.42; Group IV, 23.54 ± 1.47), lower anterior (Group III, 24.61 ± 7.14; Group IV, 23.98 ± 1.62), middle posterior (Group III, 20.75 ± 3.13; Group IV, 22. 09 ± 2.02), and lower posterior (Group III, 22.66 ± 3.39; Group IV, 23.71 ± 5.99) locations. However, no significant differences were observed among other measurement locations. In Group V, a significant difference was observed at the lower anterior (26.31 ± 9.97) location compared with the upper anterior (21.36 ± 3.00, *p* < 0.001) and upper posterior (21.84 ± 5.76, *p* = 0.04) locations (Figure 4). As to compartmental pressure measurement in different sexes, no significant differences in males compared with females were observed among different measurement locations (Figure 5).

## 4 Discussion

This research aimed to compare compartmental pressure variations among healthy volunteers of different ages, and try to verify the hypothesis about the redistribution ability of normal fascia. The compartmental pressure was only significantly higher in older subjects than in younger subjects in the upper anterior and upper posterior superficial fascial compartments. Furthermore, the results showed that the compartmental pressure at the different measurement sites was similar in each age range. However, most pressure measurement results were comparable among the different age groups and measurement locations, indicating that the fascia in healthy individuals forms a complete and interconnected cavity, which may contribute to the redistribution of local pressure increases caused by fracture, inflammation, and other diseases. These results enrich the understanding of the compartmental pressure in healthy individuals and suggest that the fascia may play a key role in pressure redistribution [14].

### 4.1 Compartment Pressure Redistribution: Fascial Structural Basis

The reason why the fascia can release or redistribute compartmental pressure is closely related to its own structure. The reason was as follows: First, fascia consists of irregularly woven connective tissue and is a highly complex, dynamic structure that can create multiple interfaces where shear strain occurs when the fascia is either passively moved due to an external force or actively moved by muscle contractions. Pathological processes, including chronic low‐back pain and scarring, can result in layers adhering to each other and reduce the maximum shear strain of the fascia [15]. Second, various cell types are present in the extracellular matrix of fascia, including glial cells and macrophages; however, the main cells in fascia are fibroblasts, and it has been found not only that the expression of matrix metalloproteinases, which are important enzymes for collagen decomposition, is increased in the early stage of ACS but also that macrophages polarize to the M2 phenotype in the later stage, which is an important sign of cell proliferation, tissue repair, angiogenesis, and phagocytosis to downregulate inflammation and eliminate waste after inflammatory events [16, 17]. Therefore, the fascia, as an important structure, may redistribute pressure through the hydrolysis and repair of collagen. Third, it has been reported that the deep muscular fascia is richly innervated with small‐diameter afferent fibers that can transmit nociceptive signals, facilitating proprioception and interoception [18]. Histological analyses have also confirmed the existence of Ruffini corpuscles [19, 20, 21], Pacinian corpuscles [19, 20, 21], and free nerve endings [22, 23]. Therefore, the fascia contributes to nonpainful sensations such as those experienced during deep pressure and stretching, but the presence and extent of the relationship between pressure transduction or release and sensation remain unknown, and further study is needed to determine the specific potential underlying mechanism [18, 23, 24]. A comprehensive understanding of the structure and function of the fascia would be helpful to clarify the potential role of fascial mobility in fascial pressure release.

### 4.2 Compartment Pressure Redistribution: Biomechanics of Fascial Mobility

Numerous well‐established and newly discovered biomechanical features of connective tissue have explained how fascial hyper‐ or hypomobility influences myofascial pressure release. When a muscle contracts and begins to transmit a load to an adjacent tendon, an initial deformation (strain) will occur before it fully bears the load, and the amount of strain will depend on the stiffness of the fascia. Furthermore, it is considered that a substantial percentage of the force exerted by a muscle is transmitted not only to the tendon but also to adjacent muscles through the fascia when the muscle contracts normally [19]. This suggests that before a force can be transmitted, the fascia is mechanically coupled in terms of shear. However, when the fascia is loose or hypermobile, force might not be transmitted to the fascia if the stress–strain curve is still in the toe region. Furthermore, if the fascia is stiff or hypomobile, adjacent tissues will become mechanically coupled sooner, and the muscles will lose a substantial amount of independent movement capability. Another important consequence is that if shear strain between layers of fascia is reduced, the layers can adhere and lose mobility altogether. Therefore, myofascial force transmission may vary depending on whether a person's connective tissue is hypermobile (younger people) or hypomobile (older people). Furthermore, unlike normal muscle contraction, fascial load bearing persists for a long time in patients with suspected ACS, and clarifying or revealing the mechanism of fascial decompression is likely to be the most important task to further support the myofascial release law hypothesis proposed in our previous research. It has been reported that the fascia in the lower limb is thicker and more mobile in younger adults than in older adults, and these characteristics may play an important role in the mechanics of locomotion [25, 26]. Furthermore, our research showed that the compartmental pressure was lower at some measurement locations in younger healthy subjects than in older subjects. Therefore, younger healthy subjects may be more inclined to recover from increased compartmental pressure without considering other factors, such as the injury mechanism and muscle volume changes.

### 4.3 Fascia Is a Potential Pressure‐Regulating Organ

Measurement of the compartmental pressure has always been regarded as the gold standard for the evaluation of patients presenting with possible ACS, which is considered to occur after the compartmental pressure increases beyond the threshold. However, it was found that with the appearance of blisters, the compartmental pressure decreased, and it was boldly proposed that fascia, as a contained organ, could play a certain role in the process of compartmental pressure release. Previous literature has indicated that fascia can play a key role as an organ and serve multiple functions. Guimberteau reported that fascia is a structure that evolves hierarchically during the development of a single‐cell embryo into an organism and that it is constantly adapting to new stresses to meet the organism's structural and functional demands [27]. Gallegos and colleagues found that fascia is beneficial and very important for wound healing, and Taguchi illustrated that deep fascia is important not only for nociception but also as a treatment target in myofascial pain patients [28, 29]. It is now clear that at least the deep muscular fascia and aponeuroses are richly innervated with small‐diameter afferent fibers that can transmit nociceptive signals [23, 24, 30]. In addition, this study also found good consistency among the pressure values measured at different compartmental locations, which confirms that the fascia is a self‐contained compartment. In turn, this characteristic enables the redistribution of pressure increases within the fascial compartment and likely helps to mitigate or even prevent the occurrence of ACS to some extent. However, when crush syndrome occurs, the normal pressure release process cannot be completed due to the extensive destruction of fascial integrity, which leads to ischemic necrosis of the contents of the compartment, including muscles. However, in fracture patients, the normal pressure release process can be completed because the fascia is almost intact; thus, it is reasonable to believe that fracture patients are not prone to ACS. The results of this study confirm that the compartmental pressure in healthy individuals is similar at different measurement points, although the upper anterior pressure was low compared with that at other locations. One reason for the increased pressure at the upper location could be proximity to the knee joint providing more space to redistribute the pressure; another potential reason for this result is insufficient rest after walking in an upright position, leading to the measurement result itself being lower than the pressure at the lower locations. Therefore, the measurement results of this study support the concept of the myofascial release law, which indicates that fascial compartment syndrome does not exist in fracture patients; however, more data are needed to support this hypothesis.

### 4.4 The Icare Pressure Monitoring Holds Extensive Application Prospects

As we mentioned, the measurement results will be affected by many factors such as muscle contractions, especially invasive modalities. Noninvasive method monitoring the muscle compartments in suspected cases becomes a very important method to minimize relative factors which affect the accuracy of compartmental pressure monitoring. There are numerous scientific researches trying to detect the increasing intra‐compartmental pressure noninvasively. For example, a noninvasive ultrasound‐based method has been widely used to detect and objectify muscle compartment elasticity for monitoring [31]. However, the ultrasound devices were so large that they are still not convenient to use in clinic. There are also other devices to measure alterations in tissue oxygen saturation, but the convenience still needs to be improved [32]. The iCare, which calculates biomechanical properties based on the tissue's inherent response (hardness of tissue) is widely used in clinic, but was first testified on skin, especially for ACS. With its advantages of portability and rapid measurement, it can be effectively employed in emergency fracture patients. It enables timely detection of abnormal intracompartmental pressure, thereby facilitating prompt interventions to prevent the onset of ACS.

### 4.5 Limitation and Strength

The key strength of this study lies in considering fascia as an organ capable of playing a pivotal role in redistributing and releasing compartmental pressure. Nevertheless, one limitation of this research was the small number of enrolled subjects; more healthy volunteers, especially older volunteers, will be included in future work. Second, the potential function and structural characteristics of the fascia were not fully revealed in this research, and the effect of muscular atrophy in older subjects compared with younger subjects on the results was not fully discussed. Third, the devices used in our research were not testified by animal model. In our future work, the compartmental pressure of patients with tibial plateau fracture and healthy subjects will be compared, and the pressure will be continuously monitored for a few days in an attempt to clarify the pattern of pressure changes in fracture patients and elucidate the relationship between blisters and compartmental pressure changes.

## 5 Conclusion

The measurement results in this study found that the fascial compartment is a self‐contained cavity with comparable pressure among most measurement locations and age groups in healthy individuals. This study proposes a new research direction for studying the fascial compartmental pressure release mechanism. The results are beneficial for decreasing the incidence and incorrect diagnosis rate of ACS and raising doubts regarding the feasibility of fasciotomy for fracture patients whose fascia is mostly intact.

## Author Contributions

**Jialiang Guo:** writing – original draft, project administration, data curation, investigation. **Jianfeng Zhang:** software. **Kezheng Du:** methodology. **Bo Shi:** methodology. **Weichong Dong:** formal analysis, project administration, resources. **Yingze Zhang:** conceptualization, supervision, validation, writing – review and editing, funding acquisition. **Zhiyong Hou:** visualization, writing – review and editing, validation, conceptualization, funding acquisition.

## Ethics Statement

Ethics approval was received from the Regional Ethics Committee of the Third Hospital of Hebei Medical University, and the study was conducted in accordance with the Declaration of Helsinki.

## Consent

Informed consent was obtained from all individual participants included in the study.

## Conflicts of Interest

The authors declare no conflicts of interest.

## Data Availability

The datasets used and/or analyzed during the current study available from the corresponding author on reasonable request.
